# Molecularly Imprinted Membranes: From Protein Recognition to Refolding Activity

**DOI:** 10.3390/polym18121482

**Published:** 2026-06-12

**Authors:** Norma Mallegni, Niccoletta Barbani, Dawid Rossino, Francesca Cicogna, Caterina Cristallini

**Affiliations:** 1 Institute of Chemistry of Organometallic Compounds, ICCOM, National Research Council of Italy (C.N.R.), 56126 Pisa, Italy; francesca.cicogna@cnr.it; 2 Institute for Chemical–Physical Processes, IPCF, National Research Council of Italy (C.N.R.), 56126 Pisa, Italy; dawidrossino@cnr.it (D.R.); caterina.cristallini@cnr.it (C.C.); 3 Department of Civil and Industrial Engineering, University of Pisa, 56122 Pisa, Italy

**Keywords:** molecularly imprinted membranes, protein recognition, protein refolding, phase inversion

## Abstract

Molecular imprinting is a powerful strategy for fabricating synthetic materials with selective recognition toward specific biomolecules. In this work, molecularly imprinted (MIM) membranes based on poly (ethylene-co-vinyl alcohol) (EVAL) were developed for selective protein recognition and conformational modulation using α-amylase as a model template. Membranes were prepared by phase inversion, generating porous structures suitable for mass transport and adsorption. Template extraction, measured using UV–Vis spectroscopy, showed a rapid and effective removal of α-amylase while preserving membrane morphology, as confirmed by SEM. FTIR-ATR and chemical imaging confirmed template removal from the membrane and a uniform surface distribution of rebound α-amylase after successive template incubation. Rebinding experiments showed a concentration-dependent uptake of α-amylase and an apparent saturation trend at higher concentrations. Selectivity tests using bovine serum albumin as an analog confirmed preferential recognition of α-amylase. Enzymatic assays showed partial recovery of catalytic activity after rebinding of thermally denatured α-amylase, indicating that imprinted cavities may promote protein conformational reorganization. These results highlight the potential of EVAL-based imprinted membranes as biomimetic platforms for selective protein recognition and functional modulation.

## 1. Introduction

Protein Conformational Disorders (PCDs) represent a class of pathologies characterized by an altered protein folding process that leads to the formation and accumulation of insoluble fibrillar aggregates of amyloid nature in tissues and organs [1]. These conditions include major neurodegenerative diseases such as Alzheimer’s disease and Parkinson’s disease, as well as systemic amyloidosis, a group of diverse disorders caused by misfolded proteins that aggregate into insoluble fibrils and ultimately cause organ damage [2]. A common feature of these disorders is the presence of amyloid deposits stabilized by serum amyloid P protein (SAP), a circulating glycoprotein capable of binding to amyloid fibrils and protecting them from proteolytic degradation [3]. The selective removal of circulating pathogenic protein species represents a promising therapeutic strategy. Conventional therapeutic plasmapheresis techniques enable the elimination of plasma components but are intrinsically poorly selective and require plasma replacement, resulting in limitations in terms of efficiency and selectivity. Therefore, the development of membrane systems with high molecular selectivity is required, capable not only of recognizing and removing specific target proteins but also of interacting with them in a functionally controlled manner. In this context, the development of selective membranes able to recognize specific protein biomolecules represents an alternative and potentially more targeted approach compared to traditional separation methods based exclusively on size cut-off or global physicochemical properties [4,5,6].

Polymeric membranes, widely employed in industrial and biomedical separation processes, allow modulation of mass transport through diffusive or convective mechanisms depending on their structure and morphology [4,5,6]. Among the preparation techniques, phase inversion represents one of the most versatile methods for the production of symmetric or asymmetric microporous membranes. Control of the thermodynamic and kinetic parameters of the process enables modulation of the formation of the superficial skin layer and the underlying porous structure, directly influencing transport properties and mechanical stability of the membrane [7,8,9].

In recent years, increasing attention has focused to the development of advanced membrane systems with enhanced selectivity, aimed at overcoming the intrinsic limitations of conventional separation processes based solely on size exclusion or bulk physicochemical properties [10,11]. In particular, the incorporation of molecular recognition elements has led to the emergence of molecularly imprinted membranes (MIMs), which enable selective interactions with specific target molecules through affinity-based mechanisms rather than purely diffusive transport [12,13].

Among the various polymers employed for biomedical membrane applications, poly (ethylene-co-vinyl alcohol) (EVAL) has attracted considerable attention due to its favorable physicochemical properties. EVAL is a synthetic copolymer composed of ethylene and vinyl alcohol units, whose composition can be tailored to modulate hydrophilicity, mechanical performance, and permeability characteristics. Importantly, EVAL has been widely investigated for blood-contacting applications [14], including hemodialysis membranes and leukodepletion filters [15], owing to its documented biocompatibility and hemocompatibility. Its balanced hydrophilic–hydrophobic character, presence of hydroxyl functionalities enabling further surface modification, and good film-forming ability further support its suitability as a matrix for molecular imprinting-based membrane systems.

Parallel to the development of separation technologies, molecular imprinting has enabled the introduction of selective recognition sites within polymeric matrices [16]. The molecular imprinting principle is based on the formation of cavities complementary in shape and functional groups to a template molecule, which is subsequently removed, leaving a stable molecular memory within the material [17]. The integration of molecular imprinting and membrane technology has led to the development of Molecularly Imprinted Membranes (MIMs), which combine structural separation properties with molecular recognition capability [18]. MIMs obtained by phase inversion enable the simultaneous formation of the porous structure and recognition sites, entrapping the template within the matrix during polymer precipitation and generating, after extraction, specific cavities distributed throughout the material volume [16,17,18]. In such systems, selectivity is no longer governed exclusively by morphological parameters but by the specific adsorption of the target protein, making these membranes comparable to affinity or adsorptive membranes [6].

Several studies have demonstrated the effectiveness of MIMs in selective rebinding and separation processes, particularly for small molecules, where high selectivity and binding affinity can be achieved. However, their application to macromolecular systems, especially proteins, remains significantly more challenging due to steric hindrance, diffusional limitations, and structural instability of the template molecules [12,19].

Despite the extensive literature on the selective rebinding capability of MIMs [16,17,20], the possibility that imprinted sites may influence the conformational state of the target protein, beyond recognition and adsorption, remains largely unexplored. In particular, the hypothesis that conformationally complementary cavities may promote the recovery of the native structure of denatured proteins introduces an innovative perspective in the biomedical applications of imprinted materials.

In the present work, molecularly imprinted membranes based on EVAL were developed using α-amylase as a model protein. The membranes, prepared by phase inversion, were characterized from both morphological and functional perspectives by evaluating template release, extraction, and rebinding kinetics as a function of time and concentration. Furthermore, the ability of the recognition sites to promote recovery of the activity of the thermally denatured enzyme was investigated.

## 2. Materials and Methods

### 2.1. Materials

Poly (ethylene-co-vinyl alcohol) (EVAL) copolymers (EVAL 44: 44 mol% ethylene and 56 mol% vinyl alcohol), dimethyl sulfoxide (DMSO, ≥98%), 3,5-dinitrosalicylic acid (DNS), potassium sodium tartrate, sodium hydroxide (NaOH), maltose, α-amylase (1,4-α-D-glucan glucanohydrolase, from porcine pancreas) and bovine serum albumin (BSA) were all purchased from Sigma-Aldrich (St. Louis, MO, USA). All aqueous solutions were prepared using bidistilled water.

### 2.2. Preparation of EVAL Membranes

#### 2.2.1. Non-Molecularly Imprinted Membranes (NMIM)

A first class of non-imprinted EVAL-based membranes (NMIM) was prepared and used as a control. EVAL was dissolved in DMSO on a heated magnetic stirrer at 100 °C until a clear and homogeneous solution was obtained (typically 1–2 h). Polymer solutions (EVAL/DMSO, 15 wt.%) were prepared by dissolving 1.5 g of EVAL in 10 mL of DMSO.

The polymer concentration of 15 wt.% was selected after preliminary membrane preparation trials performed at different EVAL concentrations. This composition provided the best balance between solution processability, homogeneous film formation, mechanical integrity after phase inversion, and formation of a porous morphology suitable for protein transport. Membranes were fabricated by inversion phase using a continuous film casting device (doctor blade) equipped with a movable casting knife (Separem Type SP, Separem, S.p.A., Biella, Italy). The first step is to pour the EVAL solution onto a glass support positioned on the horizontal casting stage. Film thickness was controlled by adjusting the casting gap (400 μm). The blade moved horizontally at a speed of 1 m/min to ensure uniform spreading of the solution. Immediately after casting, the film was immersed in a water coagulation bath to induce phase inversion. All membrane preparations were performed under standardized conditions, including constant polymer composition, casting gap, casting speed, and membrane dimensions, to ensure reproducibility of membrane morphology and template entrapment. The coagulation bath was periodically replaced over a 24 h period to ensure complete solvent removal. After coagulation, membranes were lyophilized (Christ Alpha 1–4, Osterode am Harz, Germany).

#### 2.2.2. Molecularly Imprinted Membranes Towards α-Amylase (MIM)

The procedure used for the preparation of molecularly imprinted membranes (MIM) was the same as that used for NMIM, except that an aliquot of an α-amylase solution was added to the polymer solution (EVAL/DMSO) under continuous stirring to obtain a homogeneous casting mixture. The enzyme solution was prepared by dissolving 100 mg of α-amylase in 1 mL of bidistilled water, then adding DMSO in a controlled proportion to reach a water/DMSO ratio of 50/50 (*v*/*v*), to avoid enzyme precipitation during mixing. A volume of 4 mL of polymer solution containing α-amylase (protein content equal to 0.62 wt.% with respect to the total solute) was cast onto a glass plate (8.5 cm × 14 cm) using a knife apparatus. Lower template concentrations resulted in extensive enzyme loss in the phase inversion bath due to the high solubility of α-amylase in water. Therefore, a higher template concentration was selected to ensure sufficient enzyme entrapment and effective formation of imprinted recognition sites. The EVAL/α-amylase film was immersed in the water coagulation bath. The amount of the template released during phase inversion was quantified to assess enzyme loss due to its high solubility in aqueous media. Aliquots from the coagulation baths were collected after membrane formation and analyzed by UV–Vis spectroscopy (Shimadzu UV-2100, Kyoto, Japan) at 280 nm. Template concentrations in the coagulation baths were determined from the resulting calibration equation.

Finally, the membranes were dried by lyophilization and named molecularly imprinted membranes before template extraction (MIMT). Following, MIMT was subjected to the enzyme extraction to generate MIM. The extraction procedure was based on a continuous solvent flow through the membrane under controlled pressure. Membrane samples were cut to fit a circular permeability cell (effective area: 12.56 cm^2^), where hydraulic sealing was ensured using rubber gaskets. The cell was connected to an open-loop system in which bidistilled water was circulated continuously. The extraction was carried out at a transmembrane pressure of 0.5 bar and an average flow rate of approximately 30 mL/min generated by a Masterflex^®^ peristaltic pump operating (Cole-Parmer Instrument Company, Vernon Hills, IL, USA) operating at 0.6 rpm. The system included separate outlets for permeate and retentate streams, with a valve installed on the retentate side to regulate flow and pressure. Aliquots of the permeate were collected at defined time intervals and analyzed by UV–Vis spectroscopy to determine template concentration. The amount of template removed (TR mg/mg membrane), expressed as mg of protein per mg of membrane, was calculated according to Equation (1):

(1) $$TR=\frac{V\cdot C}{W}$$

where V is the total volume of solvent passed through the membrane (mL), C is the protein concentration (mg/mL), and W is the membrane weight (mg) within the effective extraction area.

MIM membranes were finally dried by lyophilization before successive characterization.

### 2.3. Physicochemical Characterization of Membranes

#### 2.3.1. Morphological Characterization

Morphological characterization of all the produced membranes was performed by scanning electron microscopy (SEM, JSM-T300, JEOL Ltd., Tokyo, Japan), after being gold-coated by sputtering.

#### 2.3.2. FTIR-ATR and Imaging Analysis

FTIR-ATR analyses were conducted using a Spectrum Spotlight FTIR (PerkinElmer, Waltham, MA, USA). Conventional spectra were collected in ATR configuration over the 4000–600 cm^−1^ range with a spectral resolution of 4 cm^−1^, averaging 36 scans for each measurement. FTIR chemical imaging analyses were performed using a PerkinElmer Spectrum One FT–IR spectrometer (Waltham, MA, USA) equipped with a Universal ATR sampling accessory and a Spectrum Spotlight 300 FT–IR imaging system operated in ‘image’ mode (PerkinElmer Spotlight 300, Shelton, CT, USA). Measurements were carried out in micro-ATR mode on selected membrane regions covering an area of 1 mm × 1 mm. Data were acquired with a spatial sampling of 25 μm per pixel using a liquid nitrogen–cooled 16-element MCT detector (InfraTec, Dresden, Germany). Each pixel spectrum was generated from 16 accumulated scans and recorded within the 4000–750 cm^−1^ interval at 4 cm^−1^ resolution. Before the sample acquisition, background spectra were recorded on a clean ATR crystal. Areas of interest were preliminarily identified using the optical microscope integrated within the instrument, after which the ATR crystal was positioned in direct contact with the selected membrane surface. Spectral datasets were processed using Spotlight software (v1.3, PerkinElmer, Waltham, MA, USA). Pre-processing included smoothing via a Savitzky–Golay algorithm. Processed datasets were used to construct infrared distribution maps, spectral similarity (correlation) maps, and band intensity ratio images to evaluate chemical uniformity across the membrane surface. An average spectrum calculated from the entire mapped region was employed as an internal reference for correlation analysis.

#### 2.3.3. Differential Scanning Calorimetry (DSC)

Thermal properties of the membranes were evaluated by differential scanning calorimetry using a DSC 7 instrument (PerkinElmer, Waltham, MA, USA). Before analysis, the instrument was calibrated using zinc (melting point: 419.5 °C) and indium (melting point: 156.6 °C, ∆H = 28.5 Jg^−1^). The samples were placed into aluminum pans, and measurements were performed under a continuous nitrogen flow over a temperature range of 30–220 °C at a heating rate of 10 °C·min^−1^. Each specimen was subjected to two consecutive heating scans, separated by controlled rapid cooling.

### 2.4. Functional Characterization of the MIM

#### Recognition and Selectivity Studies

Rebinding experiments were performed to evaluate both recognition and selectivity properties of MIM using the same permeability setup described above for the extraction procedure, circulation of the rebinding solution through the permeability cell. NMIM was also analyzed as a control.

Rebinding Kinetics (Time-Dependent Study)

Rebinding tests were carried out using α-amylase aqueous solutions at initial concentrations of 0.3, 0.4, 0.6, and 0.8 mg/mL in order to evaluate the rebinding kinetics as a function of time and protein concentration. The selected concentration range was chosen to investigate membrane rebinding behavior under dynamic conditions over a broad interval of protein concentrations, including conditions approaching saturation of the available binding sites. Experiments were conducted under identical hydrodynamic conditions (transmembrane pressure: 0.5 bar; flow rate: 0.6 rpm). The maximum duration of each experiment was 3 h, corresponding to the average duration of a hemodialysis treatment. Aliquots of permeate were withdrawn at regular time intervals and analyzed by UV–Vis spectroscopy at 280 nm. The amount of rebound protein (RP), expressed as mg of protein per mg of membrane, was calculated according to Equation (2):

(2) $$RP=\frac{\left(C_{0}-C_{t}\right)V}{W}$$

where C_0_ is the initial protein concentration (mg/mL), C_t_ is the protein concentration in permeate at time t, V is the total solution volume (mL), and W is the membrane weight (mg) within the effective rebinding area.

For each concentration tested, the partition coefficient (Kp) and the recognition was calculated according to the following equations (Equation (3)):

(3) $$K_{p}=\frac{C_{0}-C_{eq}}{C_{0}}$$

where C_0_ and C_eq_ represent the template concentration (mg/mL) before membrane contact and after equilibrium was reached, respectively, while ${Qr}_{MIM}$ and ${Qr}_{NMIM}$ represent the amount of rebound template (mg template per mg membrane) for molecularly imprinted (MIM) and non-imprinted membranes (NMIM), respectively.

Selectivity Assessment

Selectivity was assessed by performing rebinding experiments using bovine serum albumin (BSA) as an analog protein at a concentration of 0.6 mg/mL, under the same experimental conditions adopted for α-amylase. The comparative analysis between the template protein (α-amylase) and BSA allowed evaluation of the capability of the imprinted recognition sites in distinguishing between the template enzyme and a protein analog.

Protein adsorption was calculated according to Equation (2). The concentration of BSA in the permeate was monitored by UV spectroscopy at 280 nm, and the decrease in protein concentration was used to determine the amount of protein adsorbed by the membrane.

The selectivity of the imprinted membranes (MIMs) was quantified by calculating the selectivity coefficient (Ks), defined as:

(4) $$K_{s}=\frac{{RP}_{t}[\mathrm{MIM}]}{{RP}_{a}[\mathrm{MIM}]}$$

where ${RP}_{t}$ is the amount of template protein rebound (mg template/mg membrane), and ${RP}_{a}$ is the amount of analog protein (BSA) rebound under identical conditions.

### 2.5. Evaluation of Refolding Induced by MIM

#### 2.5.1. DNS Enzymatic Activity Assay

Enzymatic activity was determined by quantifying reducing sugars produced from starch hydrolysis using the DNS method. The reaction mixture consisted of: 1 mL starch solution (5 mg/mL), 1 mL phosphate buffer (pH 6.9, containing 10 mM chloride ions). After equilibration at 30 °C for 5 min, 50 μL of enzyme solution (0.3 mg/mL) was added and incubated for 10 min at 37 °C. The reaction was stopped by adding 2 mL DNS reagent (1% *w*/*v* 3,5-dinitrosalicylic acid, 30% *w*/*v* potassium sodium tartrate, 0.4 M NaOH). Samples were heated at 100 °C for 15 min, cooled to room temperature, and absorbance was measured at 595 nm. For membrane samples, membranes (1 cm^2^; 3.6 mg) were incubated in 10 mL starch solution (10 mg/mL) under identical conditions. Aliquots were withdrawn at defined time intervals and processed as described above.

#### 2.5.2. Refolding Assessment After Rebinding of Thermally Denatured α-Amylase

Thermal denaturation of α-amylase was performed by heating an aqueous enzyme solution (0.3 mg/mL) at 80 °C for 2 h under continuous magnetic stirring [21]. A solution of the pure native enzyme was freshly prepared in phosphate buffer (pH 6.9, 10 mM chloride ions), and protein concentration was verified before each experiment as a control. Enzymatic activity for both native and denatured enzymes was calculated from the amount of reducing sugars produced, using the DNS assay described in Section 2.5.1. All measurements were performed under identical incubation conditions to allow direct comparison between samples. The denatured enzyme solution was then circulated through the MIM in a closed-loop configuration to allow rebinding onto the membrane. Following the rebinding step, the membranes (MIMR) were collected and incubated in a starch solution to assess enzymatic activity recovery. Starch was used as the specific substrate of α-amylase, and the recovery of catalytic activity after refolding was assessed by quantifying the reducing sugars produced during starch hydrolysis using the DNS assay. The enzymatic activity was measured directly on the membrane-bound enzyme at predetermined time intervals, and aliquots were withdrawn and analyzed using the DNS assay. To ensure that the observed enzymatic recovery was specifically induced by imprinting and not by residual activity or nonspecific interactions, appropriate control experiments were performed. These included thermally denatured enzyme without membrane contact, native enzyme tested in the absence of membrane exposure, and denatured enzyme after contact with non-imprinted membranes (NMIM). All experiments were conducted under identical conditions and performed in triplicate.

#### 2.5.3. Enzymatic Activity Calculation and Quantitative Refolding Analysis

The enzymatic activity measured in Section 2.5.2 was quantitatively analyzed as described below. Maltose standard solutions at known concentrations were used to establish a calibration relationship between absorbance at 595 nm and maltose concentration. Enzymatic activity was calculated from maltose production according to Equation (5):

(5) $$Activity=\frac{\Delta C}{\Delta t}$$

where ΔC represents the increase in maltose concentration (mg/mL), and Δt is the reaction time interval (15 min). To quantify the amount of active α-amylase recovered after rebinding, known concentrations of native α-amylase were subjected to the same DNS assay under identical experimental conditions. This approach allowed estimation of the effective concentration of catalytically active α-amylase in MIMR samples. Refolding efficiency was expressed as the percentage of recovered activity relative to the native enzyme control and was calculated according to Equation (6). The correlation between maltose production and α-amylase concentration was assumed to be linear under the applied assay conditions and was used to estimate the equivalent amount of active enzyme.

(6) $$Refoulding\;efficiency\;(\%)=\left(\frac{{Activity}_{MIMR}}{{Activity}_{native}}\right)\times 100$$

## 3. Results

### 3.1. Morphological Analysis

**Figure 1:**
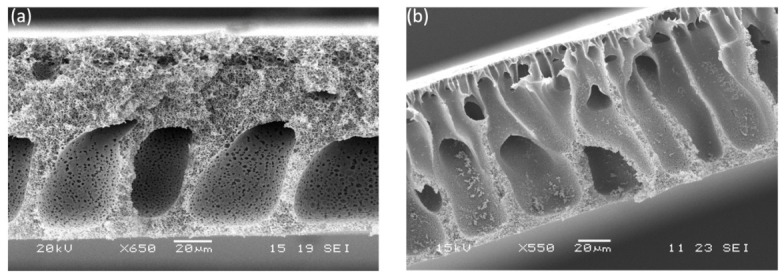
Cross-sectional SEM images of EVAL (NMIM) membranes prepared from a 15 wt.% solution in DMSO: (**a**) phase inversion in H_2_O/DMSO (50/50 *v*/*v*), (**b**) phase inversion in H_2_O.

Scanning electron microscopy (SEM) images were used to evaluate the morphology of the prepared membranes and to identify the most suitable one for the intended application. Figure 1a shows the cross-section of an EVAL/DMSO membrane prepared from a 15% polymer solution, inverted in a 50/50 H_2_O/DMSO coagulation bath for 1 h, then immersed in bidistilled water for 24 h and subsequently freeze-dried. Figure 1b, instead, reports the cross-section of an EVAL/DMSO membrane prepared using the same polymer concentration, but inverted directly in bidistilled water for 24 h and then freeze-dried.

From these images, it can be observed that the sample initially inverted in the 50/50 H_2_O/DMSO bath, before water immersion, exhibits evident porosity, particularly an asymmetric structure. In contrast, the second sample exhibits a well-defined finger-like morphology extending across the entire membrane thickness, with pore walls characterized by homogeneous microporosity. This structural difference can be attributed to the stronger nonsolvent action of pure water, which enhances solvent–nonsolvent exchange kinetics and promotes instantaneous demixing during phase inversion, favoring the formation of a well-developed finger-like morphology characterized by homogeneous micro/nanoporosity within the pore walls. Previous studies performed on analogous EVAL systems prepared under similar phase inversion conditions also demonstrated good hydraulic permeability (Lp = 4.5 × 10^−8^ cm s^−1^ Pa^−1^) and mechanical stability (elastic modulus of 2–6 MPa) under increasing pressure conditions, confirming the suitability of this porous morphology for mass transport applications [22].

**Figure 2:**
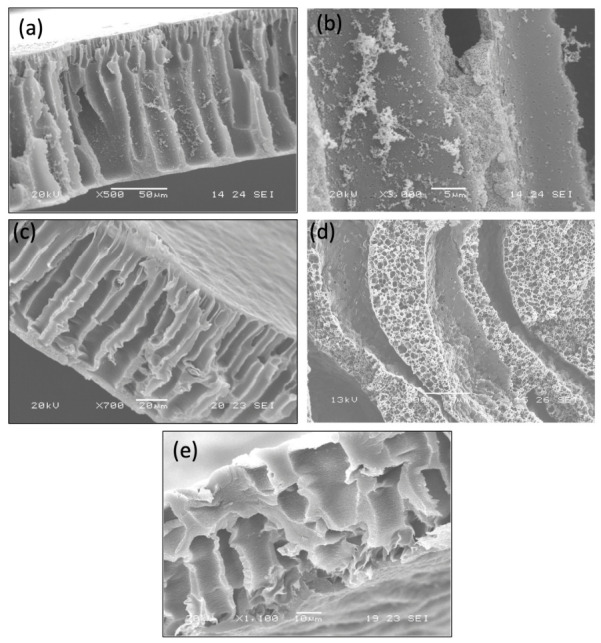
Cross-sectional SEM images of (**a**) MIMT (template-containing membrane), (**b**) higher-magnification image of MIMT, (**c**) MIM after enzyme extraction, and (**d**) higher-magnification image of MIM after template extraction, (**e**) MIMR after α-amylase rebinding. SEM images highlight the finger-like morphology and the homogeneous microporous structure of the pore walls, which is preserved after template extraction.

Such rapid phase separation favors the formation of elongated macrovoids with typical finger-like geometry, as commonly observed in diffusion-induced phase separation systems [23]. This morphology can be considered advantageous in the present system, as it promotes efficient mass transport through the membrane thickness and facilitates the diffusion of protein molecules toward the internal porous structure. The preparation procedure adopted in the second case was therefore selected, as it proved more suitable for obtaining membranes with a more homogeneous morphology. The same morphology was observed for membranes prepared in the presence of the enzyme, as shown in Figure 2a.

After a three-hour extraction treatment, SEM micrographs were acquired to evaluate possible morphology changes induced by the process. As shown in the images in Figure 2c, the porous structure remains clearly unaltered, despite the extraction procedure being relatively severe for the membrane. The preservation of the original morphology represents a clearly positive aspect in view of the subsequent rebinding experiments, as shown in Figure 2e.

### 3.2. Template Release During Inversion Bath Processing

To evaluate the actual enzyme content in MIMT, the coagulation bath was analyzed, revealing that 12.96 mg of template was released during the phase inversion process, corresponding to approximately 32.4% of the initial enzyme loading (40 mg). Consequently, about 67.6% of the enzyme remains retained within the membrane. These results indicate that, although a significant fraction of the template is lost during coagulation, a substantial amount remains entrapped within the polymeric matrix, enabling the formation of recognition sites. The interactions between the polymer and the enzyme are non-covalent. As a result, the rapid phase inversion does not permanently stabilize polymer–template interactions, but instead kinetically traps a fraction of these interactions established in the casting solution during the early stages of membrane formation. A schematic representation of the polymer–enzyme interactions and receptor site formation during phase inversion precipitation is reported in Figure S1. This enables the formation of binding cavities even if part of the template diffuses out during coagulation.

### 3.3. FT-IR and Chemical Imaging

**Figure 3:**
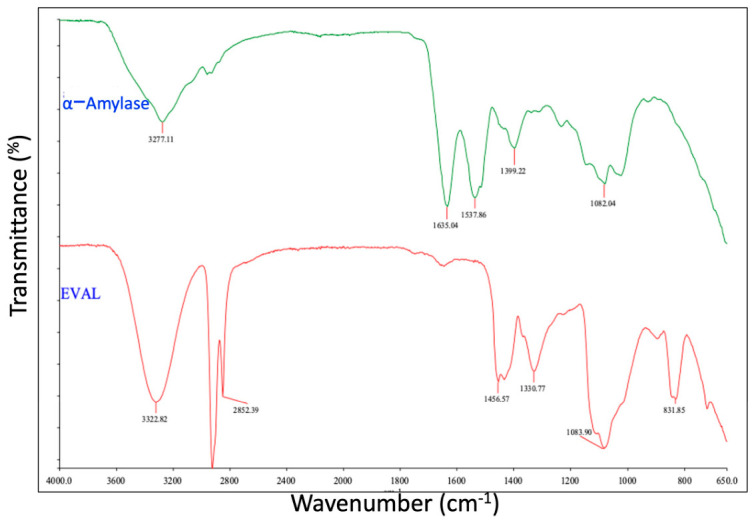
FTIR_ATR spectra of α-amylase and EVAL.

FTIR-ATR spectra of the components of our system were acquired to identify their characteristic absorption bands, as shown in Figure 3.

**Figure 4:**
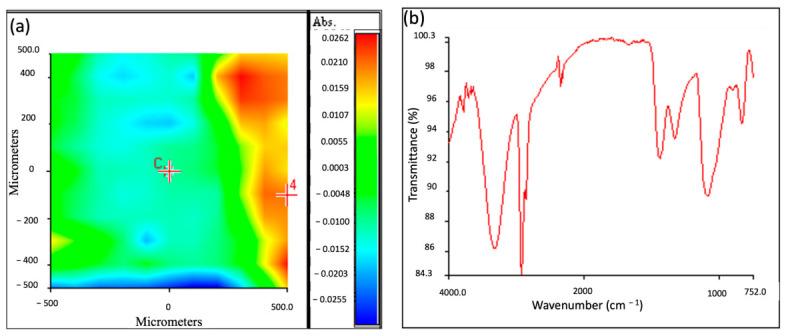
(**a**) Chemical imaging map of NMIM membrane and (**b**) corresponding characteristic spectrum.

The spectrum of α-amylase shows a broad absorption band in the 4000–3000 cm^−1^ region, attributable to the stretching vibrations of N–H and O–H groups. In addition, the characteristic amide I band at 1635 cm^−1^, associated with the C=O stretching of the peptide bond, and the amide II band at 1537 cm^−1^, related to N–H bending of the peptide bond, are clearly visible. These bands are typical of peptide bonds and are characteristic of protein macromolecules [24]. The spectrum of EVAL exhibits the characteristic absorption bands of the ethylene–vinyl alcohol copolymer. A broad band between 3500 and 3000 cm^−1^ is observed, corresponding to the O–H stretching vibrations, along with a band at 1100 cm^−1^ associated with the C–OH stretching of the vinyl alcohol units. Additional absorption bands at approximately 2900 cm^−1^ are attributed to CH_2_ and CH stretching vibrations of the ethylene component [25,26]. Chemical imaging analysis was carried out in µATR mode, which allows the evaluation of the surface chemical composition of the analyzed samples. The analysis was performed on NMIM, MIM, and MIMR membranes. The MIMR samples correspond to membranes after rebinding of α-amylase using the native enzyme for characterization purposes, whereas rebinding of thermally denatured α-amylase was specifically investigated in the refolding experiments described in Section 2.5. Figure 4 shows, respectively, (a) the chemical map of the NMIM membrane and (b) its characteristic spectrum. This spectrum displays all the absorption bands typical of EVAL.

**Figure 5:**
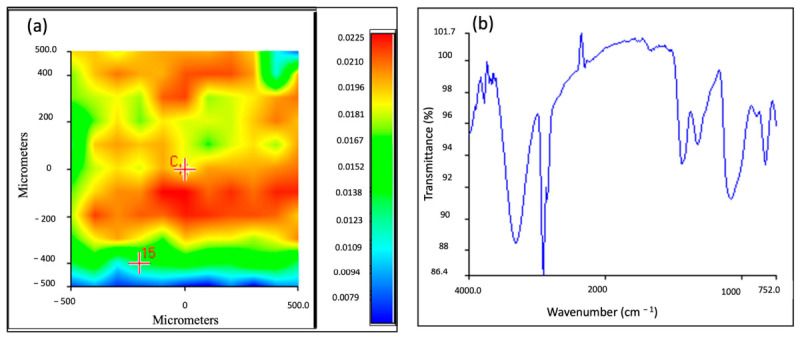
(**a**) Chemical imaging map of the MIM sample and (**b**) corresponding characteristic spectrum.

Figure 5a presents the chemical map of the extracted membrane sample and (b) its characteristic spectrum, respectively.

**Figure 6:**
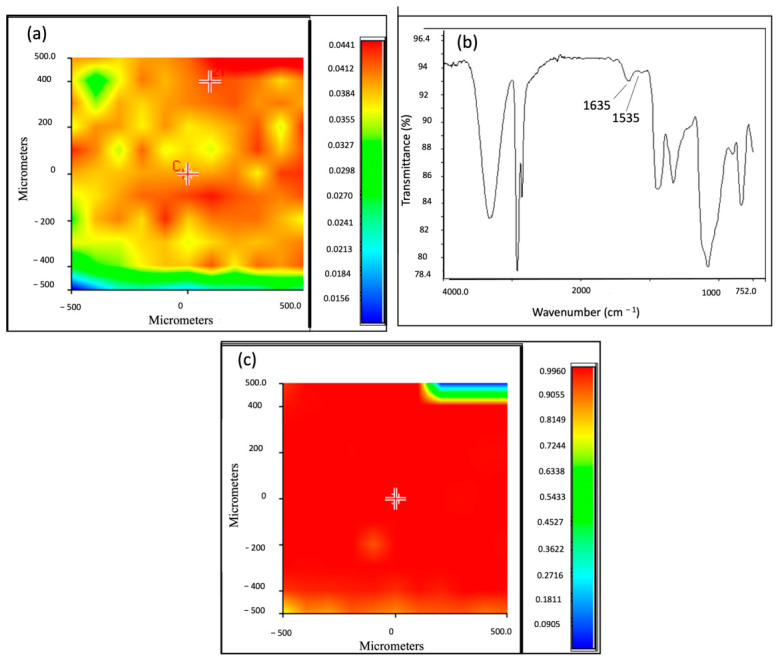
(**a**) Chemical imaging map of the MIMR membrane, (**b**) corresponding representative spectrum, and (**c**) correlation map.

For MIM, all absorption bands characteristic of EVAL are clearly observed, while no spectral features attributable to the protein are detected. This finding confirms the effectiveness of the extraction procedure in removing the enzyme from the membrane. Figure 6a shows the chemical map and (b) the characteristic spectrum of the membrane after the rebinding procedure, and (c) the correlation map. The amide I band of α-amylase at 1635 cm^−1^ was used as a diagnostic signal for chemical mapping, enabling visualization of the spatial distribution of the rebound protein within the membrane.

The characteristic spectrum of this membrane shows, in addition to the typical EVAL absorption bands, two additional absorptions at 1635 cm^−1^ and 1535 cm^−1^, corresponding to the amide I (C=O) and amide II (N–H) bands of the protein rebound to the membrane.

To evaluate the enzyme distribution on the membrane surface, a correlation map was generated, shown in Figure 6c, by comparing the reference spectrum of α-amylase with the spectra collected over the entire chemical map. High correlation values indicate regions where the spectral features match those of the enzyme, confirming its presence after rebinding. A uniform distribution of high correlation values suggests a homogeneous rebinding over the membrane surface.

### 3.4. DSC Analysis

Thermal analysis was performed on NMIM, MIMT, MIM, and MIMR membranes.

The calorimetric data obtained from the second heating scan are summarized in Table 1, and thermogram curves are reported in Figure S2. All samples exhibit a melting temperature (Tm) in the range of 160–162 °C, indicating that the crystalline phase of EVAL is preserved regardless of the imprinting, extraction, or rebinding treatments. No significant variations in Tm are observed among the different membranes, confirming that the thermal transition associated with melting remains essentially unchanged after the various processing steps. The non-imprinted membrane (NMIM) was considered as a reference, exhibiting a ΔH value of 57 J/g, representative of the native crystallinity of the EVAL matrix [27]. In the presence of the template, the MIMT membrane shows a slight decrease in the ΔH value (54 J/g), suggesting that the presence of the enzyme may influence the organization of the polymer network during membrane formation. The appearance of the shoulder in the MIMT thermogram in the 130–150 °C region may be associated with the presence of partially denatured α-amylase entrapped within the membrane during phase inversion. The enzyme–polymer interactions, mainly governed by hydrogen bonding, could locally interfere with the regular packing of EVAL chains, generating less ordered regions characterized by lower thermal stability [28,29]. This reduction can be attributed to the presence of α-amylase during membrane formation, which interferes with the regular packing of EVAL chains through intermolecular interactions, such as hydrogen bonding, thus hindering polymer crystallization [30]. After template extraction, the ΔH value increased to 62 J/g (MIM). This may suggest that removal of the enzyme does not significantly alter the structural organization of the polymer network and may support stabilization of the imprinted structure. A higher degree of crystallinity may be beneficial in molecularly imprinted systems, as it could promote the formation of a more rigid three-dimensional structure that preserves the shape and functionality of the selective recognition sites around the template molecule [12]. The rebound membrane (MIMR) exhibits a ΔH value (56 J/g) comparable to that of the non-imprinted membrane (NMIM, 57 J/g), suggesting that the amylase rebinding occurs mainly within the imprinted cavities without significantly affecting the bulk crystalline structure of the material [27]. Overall, the DSC data confirm that the melting temperature remains unaffected by the imprinting procedure, while the presence of the template molecule has a limited effect on the crystallinity of the membranes.

### 3.5. Template Extraction

**Figure 7:**
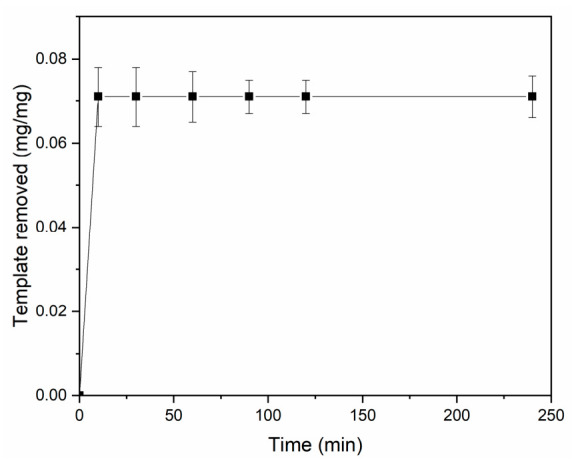
Extraction of α-amylase from MIMT membrane as a function of time. Error bars represent standard deviation (*n* = 3).

The time-dependent profile of protein removal from MIMT (Figure 7) reveals a rapid and well-defined extraction kinetics. Complete removal of the template occurs within the first few minutes of the experiment and, in any case, no later than 15 min after the start of the test.

After 15 min, the amount of extracted enzyme reaches approximately 0.07 mg of enzyme per mg of membrane. This value corresponds to the removal of nearly all the residual template remaining after phase inversion, i.e., approximately 100% of the extractable enzyme fraction. This result is consistent with the initial template loss observed during phase inversion (Section 3.2), confirming that only the fraction effectively retained within the membrane is available for extraction. These results indicate that the extraction process is highly efficient, although the presence of a minor fraction of enzyme irreversibly trapped within the polymeric matrix cannot be completely excluded. The rapid extraction kinetics can be attributed to the porous morphology of the membrane, which promotes efficient solvent penetration and mass transport throughout the structure, as previously observed by SEM analysis. Moreover, the ease of template removal is consistent with the non-covalent nature of the interactions between the polymer matrix and the protein, likely dominated by hydrogen bonding interactions as suggested by the DSC results.

### 3.6. Rebinding Tests

#### 3.6.1. Rebinding of α-Amylase

**Figure 8:**
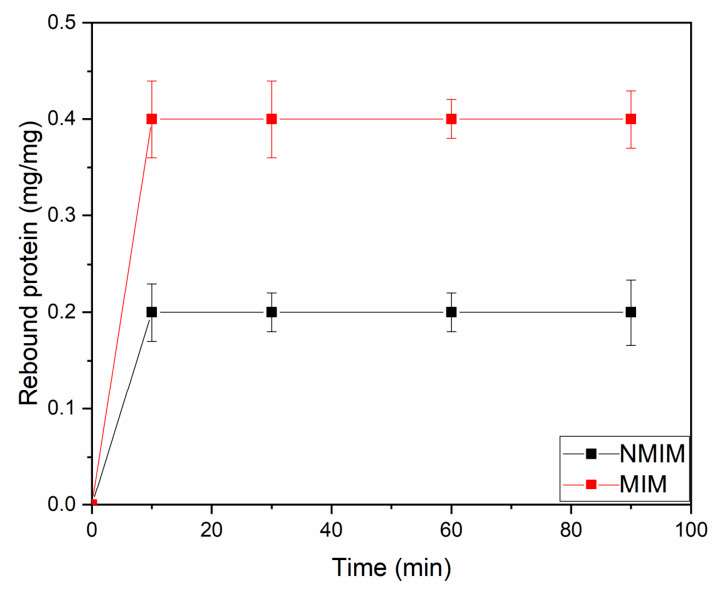
Rebinding as a function of time of α-amylase onto MIM and NMIM membranes at a rebinding concentration of 0.3 mg/mL. Error bars represent standard deviation (*n* = 3).

The time-dependent rebinding profiles of α-amylase onto MIM and NMIM membranes at an initial concentration of 0.3 mg/mL (Figure 8) clearly show a higher uptake for the imprinted membrane, indicating the presence of a specific recognition site. In particular, after the initial rapid uptake phase (within the first 15 min), both systems reach a plateau, with the MIM exhibiting an equilibrium value of approximately 0.40 mg/mg, while the NMIM stabilizes at approximately 0.20 mg/mg. In particular, the equilibrium uptake of the MIM is approximately twice that of the NMIM (approximately 0.40 vs. 0.20 mg/mg), demonstrating that nearly half of the total adsorption in the MIM can be attributed to specific binding, while the remaining fraction is due to nonspecific interactions also observed in the NMIM.

**Figure 9:**
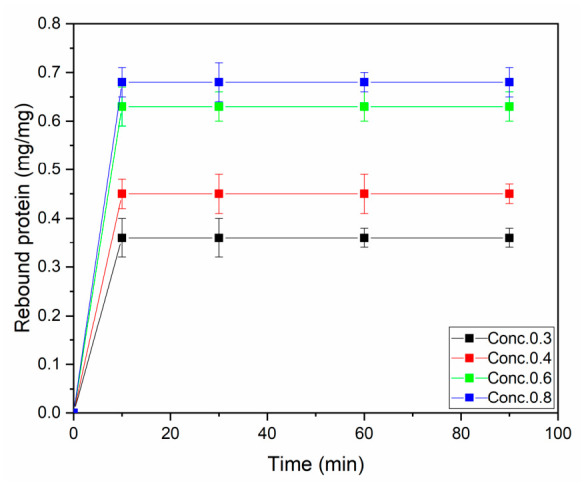
Rebinding as a function of time of α-amylase onto MIM at different initial concentrations of protein. Error bars represent standard deviation (*n* = 3).

The 0. 8 mg/mL value shows a rapid initial uptake followed by a progressive approach to equilibrium (Figure 9). For all concentrations, a steep increase in adsorption is observed within the first minutes, followed by a clear plateau, indicating that equilibrium is reached rapidly under the applied conditions. The sharp increase in adsorbed protein at short times indicates a high accessibility of imprinted regions within the polymeric matrix. This behavior indicates a high accessibility of binding sites within the polymeric matrix, likely associated with the imprinted structure and favorable mass transfer kinetics [6]. Subsequently, the curves progressively approach a plateau, indicating the attainment of adsorption equilibrium. The time required to reach equilibrium is relatively short compared to the total duration of the experiment (3 h), suggesting that the rebinding process is not significantly limited by diffusion phenomena within the porous structure [31].

Furthermore, as the initial concentration of the rebinding solution increases, the total amount of protein adsorbed at equilibrium increases correspondingly. This trend supports the presence of accessible imprinted regions contributing to selective adsorption within the polymeric matrix.

To quantitatively describe the rebinding behavior, the partition coefficients (Kp) were calculated (Table 2). The calculated partition coefficient (Kp, Equation (3)) for MIM membranes ranged between 0.17 and 0.19 across the investigated concentration interval (0.3–0.8 mg/mL), indicating a relatively constant fraction of protein adsorbed from solution. This result points to a concentration-independent uptake efficiency within this range.

The increase in the amount of rebound protein observed at higher concentrations reflects a progressive occupation of the available imprinted binding sites. Overall, these results confirm the efficient rebinding capability of the MIM membranes within the investigated concentration range.

Rebinding Isotherm (Concentration-Dependent Study)

**Figure 10:**
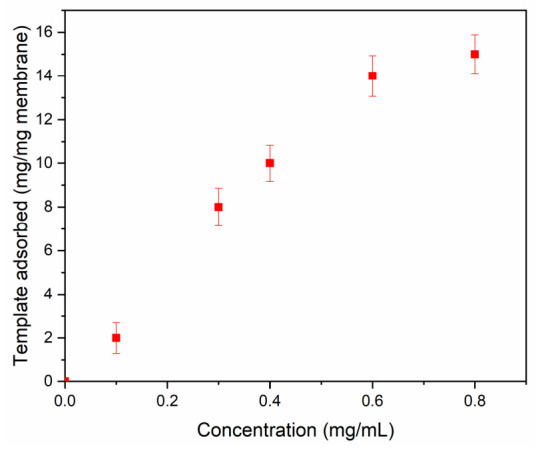
Specific rebinding isotherm as a function of template concentration. Error bars represent standard deviation (*n* = 3).

The experimental data obtained from the rebinding tests show a progressive increase in the amount of α-amylase rebound as the initial template concentration increases (0.1–0.8 mg/mL, including the additional 0.1 mg/mL data point introduced for completeness) (Figure 10). At low initial template concentrations, a nearly linear relationship is observed between the rebinding solution concentration and the amount of protein adsorbed. This behavior indicates that, within this concentration range, the recognition sites in the MIM membrane are largely available and far from saturation.

As the concentration increases, the curve gradually approaches a plateau, indicating saturation of the binding sites. At concentrations of 0.8 mg/mL, the increase in the amount of rebound protein becomes less pronounced, and only a minimal difference is observed between 0.6 and 0.8 mg/mL, indicating that the system has approached a plateau and approaches an apparent saturation regime under the investigated conditions. The same trend is observed when considering the amount of template adsorbed per unit mass of membrane, confirming the consistency of the system behavior.

#### 3.6.2. Selectivity Test, Binding of Bovine Serum Albumin (BSA)

The selectivity of the MIM toward α-amylase, used as the template for cavity imprinting in the membrane, was evaluated by rebinding experiments comparing its ability to selectively bind α-amylase and discriminate against bovine serum albumin (BSA).

**Figure 11:**
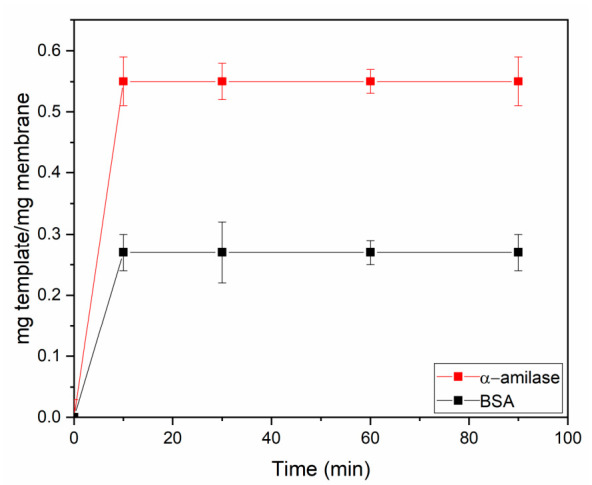
Time-dependent rebinding of α-amylase (MIM) and BSA onto MIM during the selectivity test. Error bars represent standard deviation (*n* = 3).

As shown in Figure 11, the behavior of the two proteins is markedly different. α-Amylase exhibits a rapid increase in the amount of rebound within the first minutes of contact, followed by the attainment of a stable plateau. This trend confirms the high affinity of the imprinted recognition sites toward the template protein and the good accessibility of the active sites within the polymeric matrix.

At equilibrium, α-amylase reached approximately 0.55 mg/mg, whereas BSA reached about 0.25 mg/mg, indicating that BSA adsorption is mainly nonspecific. This behavior can be attributed to surface adsorption phenomena related to the morphological characteristics of the membrane rather than to the presence of cavities complementary in shape, size, and functional group distribution.

The marked difference between the amount of α-amylase and BSA rebound demonstrates that the imprinting process successfully generated selective recognition sites with high specificity toward the template molecule.

Finally, the calculated selectivity coefficient (Equation (4)) $K_{s}=2.0$ indicates the preferential binding of α-amylase compared to BSA, supporting the effectiveness of the molecular imprinting strategy in producing selective recognition sites. The use of bovine serum albumin as a reference protein provides additional confirmation of the selectivity of the imprinted membranes. This behavior further highlights the suitability of the developed system for selective applications, minimizing undesired interactions with structurally different plasma proteins.

### 3.7. Quantitative Evaluation of Refolding Through Maltose–α-Amylase Correlation

After demonstrating the specific and selective recognition capability of the MIM, their ability to promote structural refolding of thermally denatured α-amylase was also investigated. In this section, we aim to assess whether the enzyme, inactivated by thermal denaturation, can recover its biological activity after rebinding to the specific recognition sites generated within the MIM, and to evaluate the role of the imprinted cavities in promoting the recovery of its native structure. To quantify the refolding process induced by the imprinted membrane, a correlation between maltose production and active α-amylase concentration was applied. A correlation, based on the calibration procedure, was used to relate maltose concentration (mg/mL) to the corresponding amount of active α-amylase (mg), assuming a linear trend under the assay conditions. This approach enabled the back-calculation of the effective amount of catalytically active enzyme participating in the reaction, based on the experimentally measured maltose concentration obtained from the DNS assay. The calculated values are reported in Table 3.

As expected, the native enzyme produced the highest maltose concentration (6.28 mg/mL), corresponding to 4.18 mg of active α-amylase. In contrast, the thermally denatured enzyme generated only 0.48 mg/mL of maltose (0.32 mg equivalent α-amylase), confirming the effectiveness of the denaturation treatment and the absence of residual catalytic activity. Control experiments showed that the thermally denatured enzyme without membrane contact exhibited negligible activity. Similarly, samples exposed to non-imprinted membranes (NMIM) exhibited activity values comparable to the denatured enzyme, indicating that nonspecific interactions or membrane morphology do not contribute significantly to enzymatic recovery. The MIM sample showed very low maltose production (0.2 mg/mL), corresponding to 0.13 mg of equivalent α-amylase, with values very similar to those of the denatured enzyme. This result indicates that the polymeric matrix alone does not induce significant recovery of enzymatic activity, suggesting that nonspecific membrane effects do not account for the observed activity recovery in MIMR. Notably, the MIMR sample produced 2.40 mg/mL of maltose, corresponding to 1.60 mg of active α-amylase, indicating a substantial recovery of enzymatic function after rebinding onto the imprinted membrane. The intermediate values observed for MIMR, compared to native and denatured enzyme, quantitatively suggest the occurrence of a partial refolding process induced by the specific recognition cavities generated through molecular imprinting. Based on the comparison between MIMR and native enzyme, the recovery of enzymatic activity corresponds to approximately 35–40%, confirming a refolding effect.

## 4. Conclusions

In this work, polymeric systems in the form of membranes were prepared and characterized, with the aim of recognizing protein-based biomolecules and evaluating their ability to induce protein refolding after denaturation and aggregation. This approach represents the development of a novel methodological strategy for the treatment of a class of neurodegenerative and non-neurodegenerative diseases known as Protein Conformational Disorders. The polymeric membranes were fabricated using the molecular imprinting technique combined with phase inversion. The polymer employed was the synthetic copolymer poly (ethylene-co-vinyl alcohol) (EVAL), chosen for its high hemocompatibility, thus preventing activation of the immune response. The template molecule selected was α-amylase, chosen as a model globular protein to evaluate both the selective recognition capability of the MIM system and its potential to promote recovery of enzymatic activity after thermal denaturation. The analysis of the phase inversion baths, performed by spectrophotometric quantification of the released template, demonstrated the effective entrapment of the template molecule within the membrane matrix. A key contribution was provided by infrared spectroscopy (FTIR) and chemical imaging analyses, which confirmed the presence of the template molecule, fairly homogeneously distributed within both the imprinted membranes (MIMT) and the rebound membranes (MIMR). Differential scanning calorimetry (DSC) analysis highlighted the weak nature of the interactions between the polymeric matrix and the template molecule. These interactions, mainly hydrogen bonds, are particularly advantageous, as they allow efficient extraction of the template from the polymer matrix, leading to the formation of specific cavities complementary to the template molecule. Scanning electron microscopy (SEM) revealed that all the prepared membranes exhibited a porous structure both on the surface and in cross-section. In particular, the formation of finger-like macrovoids was observed, which is characteristic of the selected phase inversion technique. A fundamental requirement for materials produced by molecular imprinting is the ability to achieve efficient and facile removal of the template molecules. This process must occur without compromising membrane integrity or its ability to undergo subsequent rebinding. Using an open-circuit permeability system and water as the extraction solvent, high levels of template removal were achieved within the first few minutes of the extraction process. Rebinding experiments demonstrated increasing amounts of rebound template as a function of the rebinding solution concentration. Although all analyzed membranes exhibited satisfactory rebinding capacity, this behavior was further confirmed by calculating typical molecular imprinting parameters, such as the partition coefficient (Kp) and the recognition factor (R). Selectivity tests conducted using bovine serum albumin (BSA) confirmed that the imprinted sites preferentially bind the template protein over other structurally similar proteins, demonstrating a high degree of specificity. Enzymatic assays performed on membranes subjected to rebinding with denatured α-amylase solutions showed that these membrane systems are capable of inducing protein refolding through molecular imprinting technology. Reusability studies based on repeated adsorption–desorption cycles will be investigated in future work. Overall, this study achieved its intended objectives, resulting in membranes characterized by high specificity toward the protein molecule used as the template. This feature endows the molecularly imprinted system with the ability to recognize and rebind harmful molecules containing specific protein units and to promote their refolding through the specific cavities generated within the molecularly imprinted membranes.

## Figures and Tables

**Table 1:** DSC data from the second heating scan.

Sample	Tm (°C)	ΔH (J/g)
NMIM	161	57
MIMT	160	54
MIM	162	62
MIMR	161	56

**Table 2:** Partition (Kp).

Sample	Protein Conc 0.3 mg/mL	Protein Conc 0.4 mg/mL	Protein Conc 0.6 mg/mL	Protein Conc 0.8 mg/mL
Kp_(MIM)_	0.18	0.19	0.19	0.17

**Table 3:** Maltose concentration and corresponding equivalent active α-amylase.

Sample	Maltose (mg/mL)	Equivalent α-Amylase (mg)
Native enzyme	6.28	4.18
Denatured enzyme	0.48	0.32
MIM	0.20	0.13
MIMR	2.40	1.60

## Data Availability

The original contributions presented in this study are included in the article and/or in the Supplementary Materials.

## Supplementary Materials

The following supporting information can be downloaded at https://www.mdpi.com/article/10.3390/polym18121482/s1, Figure S1: Schematic representation of α-amylase/EVAL interactions, Figure S2: Representative DSC thermograms of NMIM, MIMT, MIM, and MIMR membranes obtained during the second heating scan.

## Abbreviations

The following abbreviations are used in this manuscript:

PCDs	Protein Conformational Disorders
SAP	Serum Amyloid P protein
EVAL	Poly (ethylene-co-vinyl alcohol)
MIMs	Molecularly Imprinted Membranes
DMSO	Dimethyl sulfoxide
DNS	3,5-dinitrosalicylic acid
NaOH	Sodium hydroxide
BSA	Bovine serum albumin
NMIM	Non-imprinted membrane
MIMT	Template-containing membrane
MIM	Template-extracted membrane
MIMR	Rebound membrane
SEM	Scanning Electron Microscopy
FTIR-ATR	Fourier Transform Infrared Spectroscopy Attenuated Total Reflectance
DSC	Differential Scanning Calorimetry
UV-Vis	Ultraviolet–Visible Spectroscopy
Kp	Partition coefficient
Ks	Selectivity coefficient
R	Recognition parameter
Rp	Rebound protein
