# Supplementary material for: Molecularly Imprinted Membranes: From Protein Recognition to Refolding Activity
Source: Polymers (Basel). 2026 Jun 12;18(12):1482. doi: 10.3390/polym18121482 (PMC13306326; doi:10.3390/polym18121482)
Supplement: Supplementary file 1 [file polymers-18-01482-s001.zip › polymers-4331860-supplementary.pdf]

Article

Molecularly Imprinted Membranes: From Protein Recognition to Refolding Activity

Norma Mallegni [1,*,†], Niccoletta Barbani [2,3,*,†], Dawid Rossino [2,3], Francesca Cicogna [1] and Caterina Cristallini [2,3]

[1] Institute of Chemistry of Organometallic Compounds, ICCOM, National Research Council of Italy (C.N.R.), 56126 Pisa, Italy; francesca.cicogna@cnr.it
[2] Institute for Chemical–Physical Processes, IPCF, National Research Council of Italy (C.N.R.), 56126 Pisa, Italy; dawidrossino@cnr.it (D.R.); caterina.cristallini@cnr.it (C.C.)
[3] Department of Civil and Industrial Engineering, University of Pisa, 56122 Pisa, Italy
* Correspondence: norma.mallegni@cnr.it (N.M.); niccoletta.barbani@unipi.it (N.B.)
† These authors contributed equally to this work.

Amylase/EVAL interactions

A schematic representation of the proposed interactions occurring during membrane formation is shown in Figure S1. During phase inversion, hydroxyl groups of EVAL may establish non-covalent interactions, mainly hydrogen bonding, with polar amino acid residues exposed on the α-amylase surface. These interactions may promote local organization of the polymer chains around the template molecule, leading, after template extraction, to the formation of complementary recognition cavities within the membrane matrix.

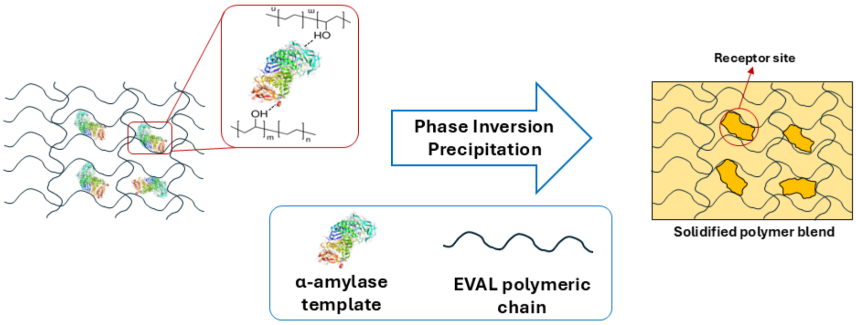

Figure S1. Schematic representation of α-amylase/EVAL interactions.

DSC thermograms

Second heating scan thermograms of EVAL-based membranes at different stages of the molecular imprinting process: NMIM, MIMT, MIM, and MIMR.

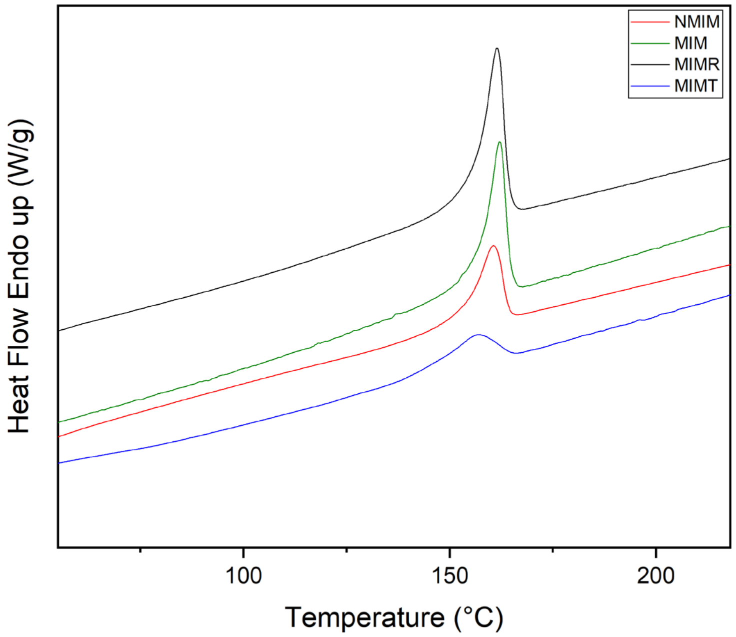

Figure S2. Representative DSC thermograms of NMIM, MIMT, MIM, and MIMR membranes obtained during the second heating scan.
